# Rapunzel Syndrome—An Extremely Rare Cause of Digestive Symptoms in Children: A Case Report and a Review of the Literature

**DOI:** 10.3389/fped.2021.684379

**Published:** 2021-06-09

**Authors:** Cristina Oana Marginean, Lorena Elena Melit, Maria Oana Sasaran, Razvan Marginean, Zoltan Derzsi

**Affiliations:** ^1^Department of Pediatrics I, George Emil Palade University of Medicine, Pharmacy, Science, and Technology of Târgu Mureş, Târgu Mureş, Romania; ^2^Department of Pediatrics III, George Emil Palade University of Medicine, Pharmacy, Science, and Technology of Târgu Mureş, Târgu Mureş, Romania; ^3^Department of Pediatric Surgery, County Emergency Clinical Hospital of Târgu Mureş, Târgu Mureş, Romania; ^4^Department of Pediatric Surgery, George Emil Palade University of Medicine, Pharmacy, Science, and Technology of Târgu Mureş, Romania

**Keywords:** Rapunzel syndrome, children, trichotillomania, trichobezoar, trichophagia

## Abstract

Rapunzel syndrome is an extremely rare condition seen in adolescents or young females with psychiatric disorders consisting of a gastric trichobezoar with an extension within the small bowel. The delays in diagnosis are common since in its early stages, it is usually asymptomatic. We report the case of a 13-year-old girl admitted in our clinic for abdominal pain, anorexia, and weight loss. The clinical exam pointed out diffuse alopecia, a palpable mass in the epigastric area, and abdominal tenderness at palpation, the patient weighing 32 kg. The laboratory tests showed anemia. The abdominal ultrasound showed a gastric intraluminal mass with a superior hyperechoic arc. The upper digestive endoscopy revealed a mass formed by hair, mucus, and food occupying the gastric cavity with the extension into the duodenum confirming the diagnosis of Rapunzel syndrome. The giant trichobezoar of 511 g, measuring 17 × 7 × 6.5 cm with a tail of approximately 3 cm, was successfully removed through laparotomy. Although rare, Rapunzel syndrome must never be forgotten as a differential diagnosis for digestive symptoms since its early detection hinders the occurrence of further complications.

## Introduction

The word “bezoar” originates from the Arabic word “bedzehr” or the Persian “padzhar,” which means “protecting against a poison,” since historically, bezoars from animal guts were useful as antidotes to poisons and, nowadays, as part of traditional Chinese medicine (1, 2). In humans, the first bezoar was described in 1779 in a deceased patient with gastric perforation and peritonitis (3). Depending on their content, multiple types of bezoars were reported: trichobezoars mostly contain hair and the most common type in humans; phytobezoars, are made of vegetable or fruit fiber; lactobezoars are made of milk curd; and pharmacobezoars involves tablets and semifluid medications, or those formed by any indigestible material (4). It is well known that human hair is resistant to digestion and peristalsis due to its smooth surface resulting in its accumulation within the stomach. Therefore, repeated trichophagia (i.e., the ingestion of hair) leads to the impaction of hair along with mucus and food within the stomach resulting in the formation of trichobezoar, which is commonly limited within the stomach. Nevertheless, in rare cases, it may present an extension through the pylorus into the small intestine or even into the colon, a condition known as Rapunzel syndrome (5).

Rapunzel syndrome was described for the first time in 1968 by Vaughan et al., and it is almost exclusively seen in young females (3, 6, 7). Multiple definitions emerged for this syndrome, but overall, it represents a trichobezoar with a tail extending in the small bowel (5). The name of this syndrome comes from a fairy tale about a 12-year-old princess who was locked in a tower without stairs or doors and managed to escape with the help of Rapunzel's long tresses (8). Young females diagnosed with trichobezoars or Rapunzel syndrome are usually associated with a psychiatric disorder, and it was reported that abuse, pica, mental disorders, depression, anorexia nervosa, or obsessive–compulsive disorder might represent potential comorbidities (9–11). The clinical picture is usually scarce in the early stages resulting in common delays of diagnosis. Thus, the awareness of trichotillomania, i.e., the urge to pull out one's own hair, associated to trichophagia, i.e., swallowing hair, is mandatory for an early diagnosis of trichobezoar in women with psychiatric comorbidity. If not recognized, trichobezoar continues to grow in weight and size occupying completely the stomach with increased risk for gastric mucosal erosions or ulcerations, and even gastric perforation (12). Moreover, parts of the trichobezoars' tail might rupture and migrate into the small intestine resulting in severe complications, such as bowel obstruction, perforation, and peritonitis in advanced cases (12). Other complications have also been reported in patients with trichobezoars, such as protein-losing enteropathy, intussusception, obstructive jaundice, pancreatitis, or even death in cases of unrecognized bezoars or delayed diagnosis (13–16).

In terms of treatment, multiple options were reported, among which are medical treatment and enzymatic degradation, endoscopic, laparoscopic, or laparotomic removal (12).

The aim of this case report was to increase awareness of Rapunzel syndrome as a rare cause of weight loss, anorexia, abdominal pain, and anemia in children.

Written informed consent was obtained from the patient's mother, the minor's legal guardian, for the publication of any potentially identifiable images or data included in this article.

## Case Report

### Presenting Concerns

We report the case of a 14-year-old female admitted in our clinic for weight loss (approximately 5 kg within the last month), anorexia, and abdominal pain. The anamnesis revealed that the patient was diagnosed with mild developmental delay without an identifiable cause despite her management by a neuropsychiatrist. The patient's socioeconomic level was appropriate, and we found no evidence of child neglect.

### Clinical Findings

The clinical exam at the time of admission pointed out diffuse alopecia, a palpable mass in the epigastric area, and abdominal tenderness at palpation. The patient weighed 32 kg.

### Diagnostic Focus and Assessment

**Figure 1:**
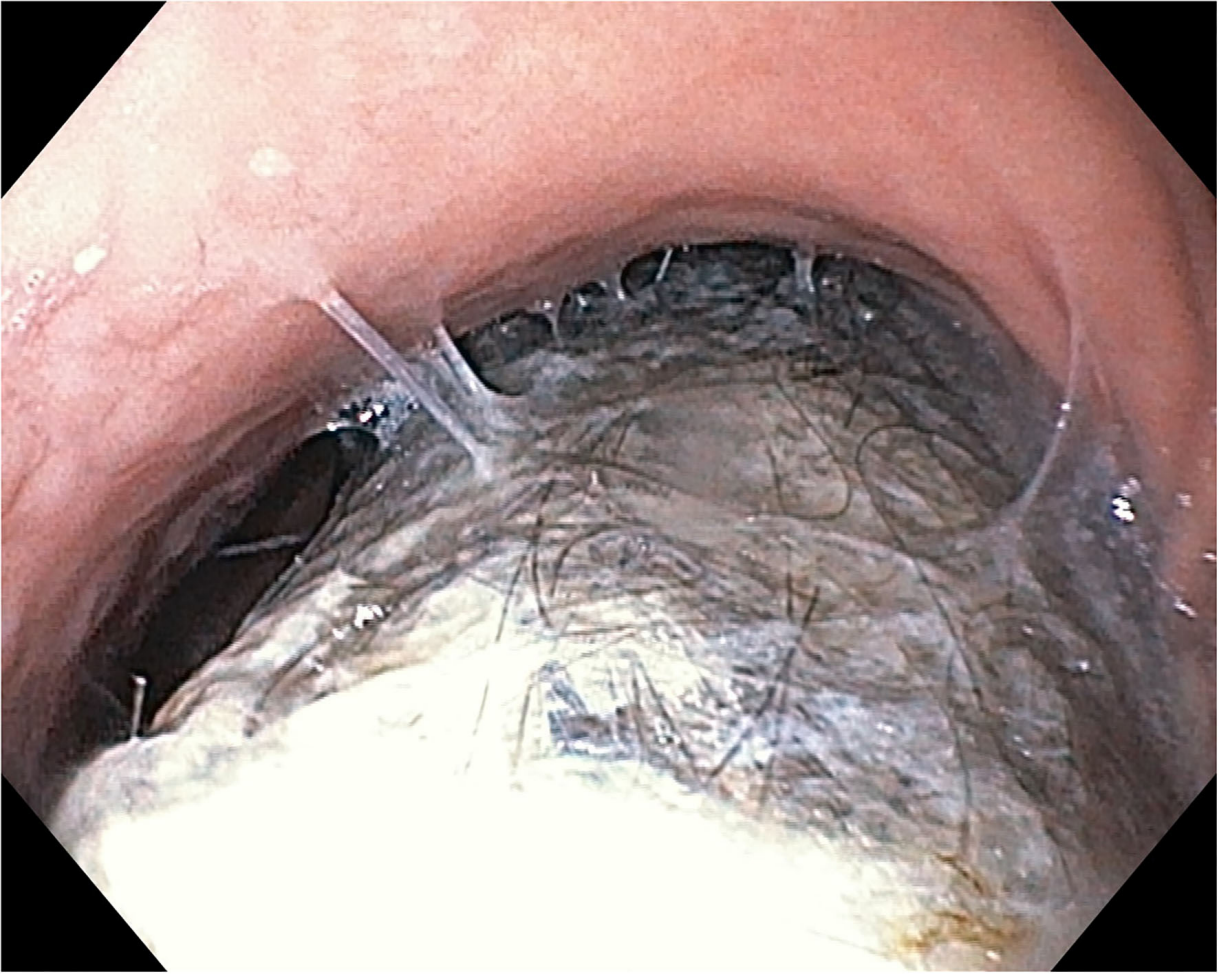
Endoscopic aspect of trichobezoar—at the level of gastric corpus.

**Figure 2:**
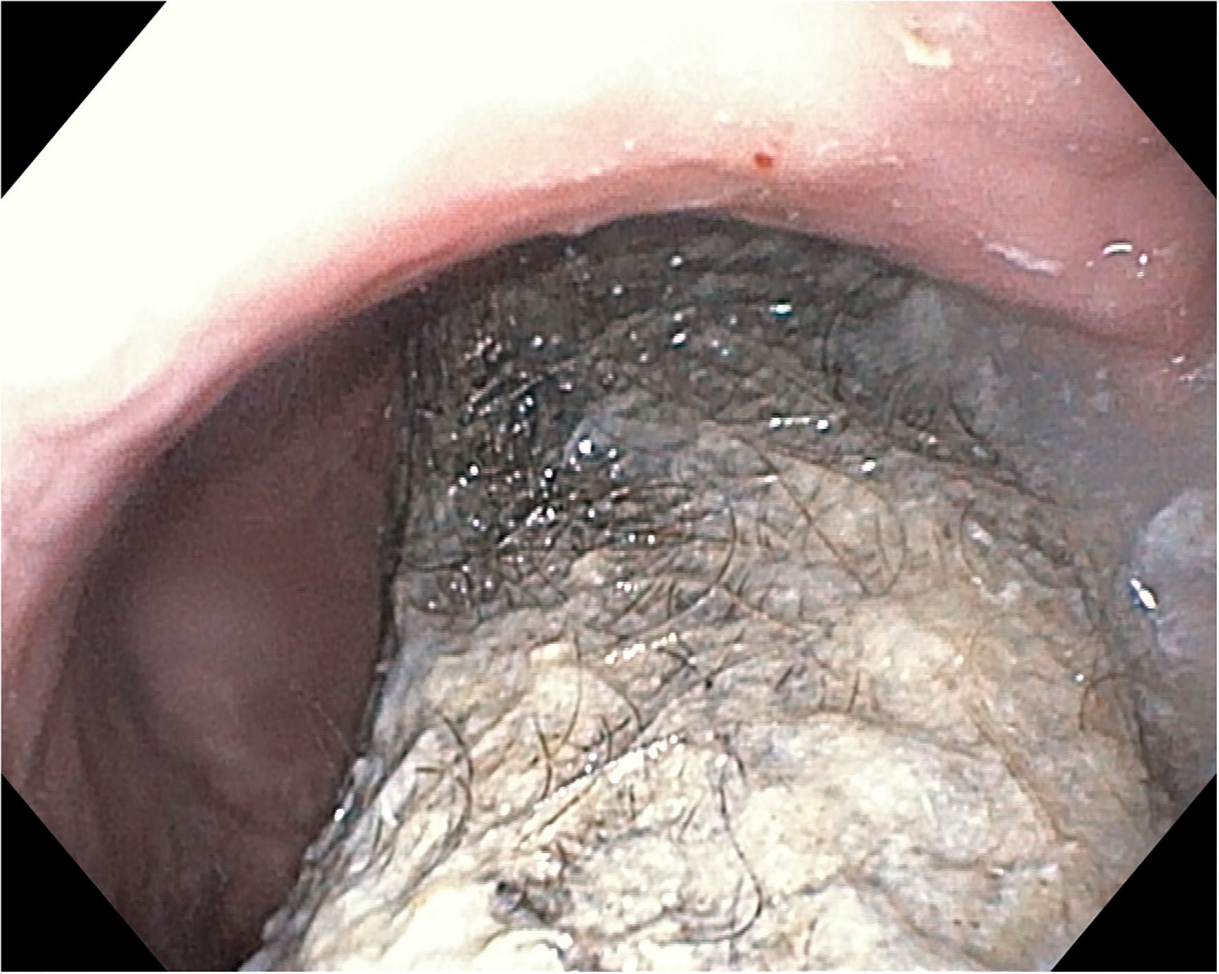
Endoscopic aspect of trichobezoar—at the level of gastric corpus and antrum.

Based on the presenting concerns and the clinical findings, we raised the suspicion of trichotillomania and trichophagia resulting in a possible trichobezoar. Initially, the patient denied to have ingested her hair, but after a thorough anamnesis, she admitted to have this habit, but rarely. The laboratory tests showed anemia (hemoglobin 8.7 g/dl, hematocrit 30.5%, and medium erythrocyte volume 67 fl). The abdominal ultrasound showed a gastric intraluminal mass with a superior hyperechoic arc. The upper digestive endoscopy revealed a mass formed by hair, mucus, and food (Figures 1, 2) occupying the gastric cavity with the extension into the duodenum confirming the diagnosis of Rapunzel syndrome.

### Therapeutic Focus and Assessment

**Figure 3:**
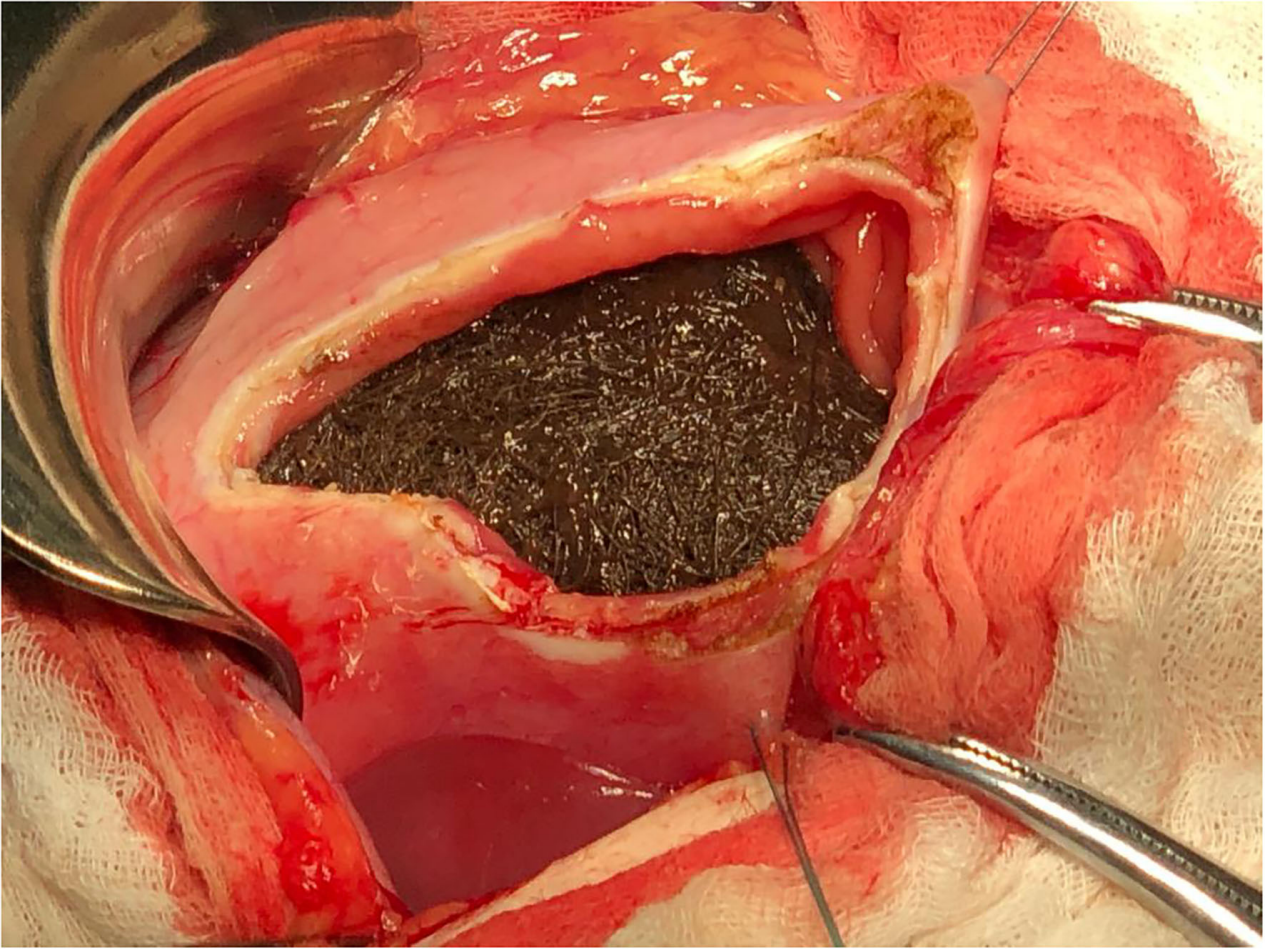
Intraoperator aspect of trichobezoar.

**Figure 4:**
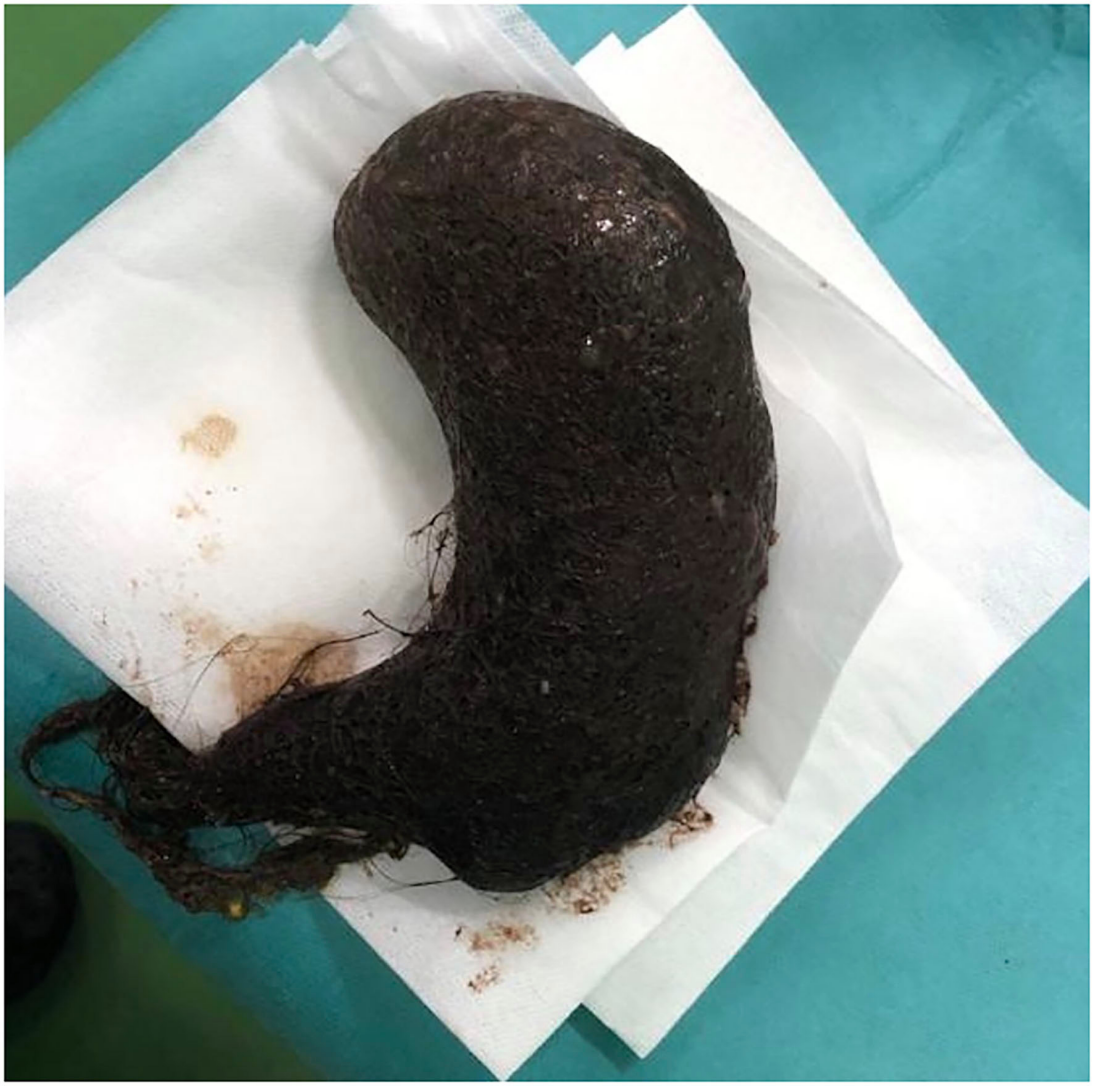
Macroscopic aspect of hole trichobezoar.

The size of this mass did not allow us to remove it endoscopically, and therefore, the patient was referred to the pediatric surgery, and the trichobezoar was surgically removed by median supraumbilical laparotomy with longitudinal gastrotomy (Figures 3, 4). We found no satellite trichobezoars in either small or large intestine. The giant stomach-shaped trichobezoar weighed 511 g, measuring 17 × 7 × 6.5 cm with a tail of approximately 3 cm.

### Follow-Up and Monitoring

The patient presented a postsurgical favorable evolution tolerating well the oral progressive feeding, without requiring nutritional support, but the main burden consists in a proper long-term psychiatric monitoring for an effective prevention of both trichotillomania and trichophagia. Thus, once she fully recovered after the surgical intervention, she was referred to the Pediatrics Neuropsychiatry Clinic where an oral treatment with alprazolam was initiated. In terms of anemia, the laboratory tests at 1 month after the surgical intervention revealed considerably improved parameters: hemoglobin 10 mg/dl, hematocrit 36.6%, and medium erythrocyte volume 70.9 fl. Nevertheless, we decided to introduce oral iron supplementation for 6 weeks.

## Discussion

Trichobezoars, the most common bezoars in humans, usually occur in preadolescent or adolescent girls with psychiatric comorbidities or developmental delay (6, 17, 18). Similarly, our case describes a preadolescent girl previously diagnosed with developmental delay. Gorter et al. described four cases of trichobezoars all in girls aged between 7 and 15 years, but Rapunzel syndrome was identified only in one case (12). Nevertheless, trichobezoars are also possible in males as reported by Hal et al. describing to the best of our knowledge the youngest patient diagnosed with trichobezoar, a 3-year-old boy (19).

Anamnesis is a key factor in raising the suspicion of hair consumption and its relevance relies especially on the physician's communication skills (20) since these patients usually refuse to provide this information even in the presence of obvious alopecia (6). Our patient also refused initially to confess that she ingested her hair, but eventually, we managed to overpass these communication barriers and determined to get her to acknowledge both trichotillomania and trichophagia. Abdominal pain, most commonly functional, is probably among the most frequent causes for pediatric gastroenterology referral. Although abdominal pain is a common symptom in patients with bezoars, this diagnosis is usually missed in children complaining of abdominal pain or other digestive symptoms most likely due to its rarity (6, 21). Among the series of cases presented by Gorter, chronic abdominal pain and weight loss were reported in two of the four cases (12). Other common presenting complains are nausea, vomiting, obstruction or peritonitis, while weight loss, anorexia, intussusception, and hematemesis were less commonly reported in patients with trichobezoars (1). Our patient complained of abdominal pain, but presented also weight loss and anorexia. Anemia is a common finding in patients with pica, but multiple controversies exist on whether pica or anemia comes first. Thus, it was emphasized that iron deficiency triggers the ingestion of clay (22), but geophagia might induce iron deficiency by reducing iron absorption (23, 24). Nevertheless, a study performed on children with sickle cell anemia found that children diagnosed with pica were more anemic compared with non-pica children (25). A recent review on this topic concluded that pica is commonly seen in children who suffer from developmental delay, and it is an important cause of anemia (26). Although the relationship between pica and anemia is not clearly defined, it is undoubtable that the treatment of anemia is crucial for the long-term outcome of children who practice pica. Similarly, our patient presented both developmental delay and anemia in association with trichophagia and received iron oral supplements.

The diagnosis is usually suspected as a result of thorough anamnesis and clinical exam, but it requires imaging tools for its confirmation. Upper digestive endoscopy is considered as the gold standard for diagnosis in trichobezoars revealing a mass formed by hair, which appears black due to the effect of the gastric acid on the hair protein, mixed with mucus and food (1, 27). We also performed an upper digestive endoscopy in our patient, which confirmed the clinical suspicion of trichobezoar, revealing also its prolongation into the duodenum. Nevertheless, the most commonly used imaging tool reported in the literature is computed tomography showing a typical well-defined intraluminal ovoid-shaped heterogenous mass with interspersed gas (28). Another useful imaging tool in establishing the diagnosis of trichobezoar is represented by upper gastrointestinal series, which will reveal a filling defect in the stomach (29). Thus, Fallon et al. assessed seven patients with trichobezoars and found that upper gastrointestinal series were performed in two of these patients revealing both the trichobezoar and its postpyloric extension in both cases (6).

The usefulness of medical treatment and enzymatic degradation is limited to small trichobezoars, but usually fail in proving their effectiveness. Studies reported that Coca Cola might be useful for the degradation of gastric phytobezoars, but not trichobezoars (30). Endoscopic removal, although attractive, has a very low success rate being reported to be effective usually in small trichobezoars (31). In our case, it was impossible to attempt endoscopic removal due to the large size of the trichobezoar occupying almost the entire gastric cavity. Laparoscopy is a relatively better alternative compared with endoscopy, but it is usually converted into open laparotomy (12). Nevertheless, the successful rate might increase as a result of the combination between laparoscopic fragmentation of trichobezoar and endoscopic removal of the fragments (32). Despite its potential complications, laparotomy was reported to be 100% effective for trichobezoar removal especially in the setting of Rapunzel syndrome (12). Our patient also underwent a laparotomy, and the giant trichobezoar along with its tail were successfully removed. The long-term prognosis of these patients depends completely on preventing recurrences. Therefore, parental counseling, behavioral therapy for controlling trichotillomania and trichophagia, as well as psychiatric and psychological support and close follow-up are essential in improving the outcome of children previously diagnosed with trichobezoars. Similarly, treatment with alprazolam was also initiated in our case after approximately 3 weeks from the surgical intervention.

## Conclusions

Rapunzel syndrome is an extremely rare cause of digestive symptoms, such as abdominal pain, anorexia, or weight loss in children. Delays in diagnosis are relatively common taking into account that it is rarely considered in the differential diagnosis of children with digestive symptoms. Increased awareness regarding risk factors for trichotillomania and trichophagia, such as developmental delay and anemia as a cause of pica, is crucial for the early diagnosis. Laparotomy remains the treatment of choice in the case of large bezoars. Thus, albeit rare, Rapunzel syndrome must never be forgotten since its early detection hinders the occurrence of further complications.

## Data Availability Statement

The original contributions presented in the study are included in the article/supplementary material, further inquiries can be directed to the corresponding author/s.

## Ethics Statement

Written informed consent was obtained from the patient's mother, the minor' legal guardian, for the publication of any potentially identifiable images or data included in this article.

## Author Contributions

CM, LM, and MS conceptualized and designed the study, drafted the initial manuscript, and reviewed and revised the manuscript. CM performed the gastroscopy. MS designed the data collection instruments, collected data, carried out the initial analyses, and reviewed and revised the manuscript. ZD and RM performed the surgical intervention. All authors approved the final manuscript as submitted and agreed to be accountable for all aspects of the work.

## Conflict of Interest

The authors declare that the research was conducted in the absence of any commercial or financial relationships that could be construed as a potential conflict of interest.
